# Snout Shape in Extant Ruminants

**DOI:** 10.1371/journal.pone.0112035

**Published:** 2014-11-05

**Authors:** Jonathan P. Tennant, Norman MacLeod

**Affiliations:** 1 Department of Earth Science and Engineering, Imperial College London, London, United Kingdom; 2 Earth Science Department, The Natural History Museum, London, United Kingdom; Raymond M. Alf Museum of Paleontology, United States of America

## Abstract

Snout shape is a prominent aspect of herbivore feeding ecology, interacting with both forage selectivity and intake rate. Previous investigations have suggested ruminant feeding styles can be discriminated via snout shape, with grazing and browsing species characterised by ‘blunt’ and ‘pointed’ snouts respectively, often with specification of an ‘intermediate’ sub-grouping to represent ambiguous feeding styles and/or morphologies. Snout shape morphology is analysed here using a geometric morphometric approach to compare the two-dimensional profiles of the premaxilla in ventral aspect for a large sample of modern ruminant species, for which feeding modes are known from secondary criteria. Results suggest that, when browsing and grazing ruminants are classified ecologically based on a range of feeding style indicators, they cannot be discriminated unambiguously on the basis of snout profile shape alone. Profile shapes in our sample form a continuum with substantial overlap between groupings and a diverse range of morphologies. Nevertheless, we obtained an 83.8 percent ratio of correct post hoc feeding style categorisations based on the proximity of projected profile shapes to group centroids in the discriminant space. Accordingly, this procedure for identifying species whose feeding strategy is ‘unknown’ can be used with a reasonable degree of confidence, especially if backed-up by additional information. Based on these results we also refine the definitions of snout shape varieties, taking advantage of the descriptive power that geometric morphometrics offers to characterize the morphological disparities observed. The shape variance exhibited by both browsing and grazing ruminants corresponds strongly to body mass, providing further evidence for an interaction between snout shape, feeding style, and body size evolution. Finally, by exploring the role of phylogenetic similarity in snout shape, we find a slight increase in successful categorisation when repeating the analysis with phylogenetic control on the geometric profiles.

## Introduction

Members of Ruminantia are even-toed ungulate mammals defined uniquely by possession of a two-step digestion system involving the fermentation chamber in the foregut of the stomach. Some 200 extant species currently recognised [1]. Ruminant feeding styles are reflected in their craniodental and gastrointestinal morphophysiological diversity, and have been conventionally categorised into ‘browsers’ and ‘grazers’, with an ‘intermediate’ sub-category [2]–[5]. Additionally, browsers are considered obligate non-grazers, but not vice-versa [2]. Some authors further include variants of frugivores, high-level browsers, and fresh grass grazers as independent categories in an attempt to encompass a larger range of feeding styles [3]–[5]. Variations in feeding style may also occur on different spatial and temporal levels, corresponding to environmental stresses (e.g., drought [6]), and plausibly a hierarchical grazing succession related to species’ migration patterns, geomorphology, resource partitioning or forage quality [7]–[9].

Van Zyl was the first to define an ecological classification scheme for ungulates based explicitly on feeding style [10]. Hofmann extended Van Zyl’s definitions to contain a novel qualitative morphological and physiological underpinning [11]–[16]. Hofmann’s modified ungulate feeding classification scheme has been used extensively in vertebrate (palaeo)biology ever since. Recently, availability of software, new data-analysis techniques, and increased computational power have combined to facilitate the use of a wider range of approaches, including geometric morphometrics, that allows us to build on and re-appraise these earlier findings (see Clauss *et* *al*. [17] and references therein).

Despite a range of morpho-behavioural correlates, the archetypal dichotomy between ‘browsers’ and ‘grazers’ is based on a botanical foundation. Browsers typically consume dicotyledonous leaves, stems, twigs and fruits [11], [18], [19]. Grazers consume monocotyledonous plants, and ‘intermediate’ feeders vary their consumption preferences depending on season and geography [20], [21]. The putative morphological significance of this dietary variation is that the physical, mechanical and biochemical properties of different forage types are adequate to drive and maintain a morpho-functional trichotomy among ruminant species that reflects the physical challenges they face accessing and/or processing different types of forage. It has been argued that the biomechanical properties of different forage types have exerted strong controls on the evolution of the masticatory apparatus and gastrointestinal tract [2], and specifically the reticulorumen physiology [22], [23] within ruminants.

The botanical definitions underpinning the classification of browsers and grazers have a complex history, with technical articles containing numerous examples of inconsistent thresholds for distinctions between these classes. Several authors have regarded browsers to be ruminants that consume less than 10 percent grass, and grazers to be those consuming greater than 90 percent grass per annum, with all other species classed as intermediate [24]–[27]. Conversely, others have selected greater than 75 percent grass consumption per annum as the threshold criterion for their grazer class, and greater than 75 percent browse consumption for the browser class, with little or no empirical evidence or logical rationale provided for these thresholds [4], [28]–[30]. Clauss *et* *al*. [31] defined grazers as those ungulates consuming greater than 80 percent monocot material, and strict browsers as those with a “very low intake of monocot forage” (p. 399) based on a small sample, similar to a range of comparative studies in which percentage of grass consumed is used as the basis for further investigations into ruminant ecology [2], [17], [23], [32].

In other studies, feeding strategy delimitation has been based purely on qualitative assessments [33], where grazers are classified as those “consuming primarily grasses, sedges and other graminoids” (p. 178). This discordant usage has been summarised partially by Clauss *et* *al*. [17], with Gordon and Illius [34] finding that different thresholds of classification give different results in ecological analyses. Accordingly, defining these thresholds in congruence with functional or ecological significance remains a problematic issue and one which is only exacerbated when such thresholds are used as a basis for further investigation of ruminant ecology. Additionally, species-level variation in diet and the physiology and morphology of ingestion due to the facultative nature of feeding strategies makes placing discrete parameters around distinct ecological sub-groups challenging [35].

There are numerous morphophysiological parameters that might, in principle, affect digestive rates and masticatory efficiency among ruminant species. Among these, the anterior snout forms the interactive part of the ingestive apparatus that interacts with any and all types of ruminant forage [36]. The anterodorsal section of the snout is formed predominantly by the premaxillae. It has been noted commonly that browsing ruminant species have pointed snouts and grazers a more squared or blunt shape representing a derived cropping condition (e.g., [24], [37]). Intermediate feeding styles have been posited to have an intermediate form, considered to conform to a mediolaterally compressed club-like shape [38].

The relationships between the various aspects of herbivore ingestion are complicated, but recent developments have provided considerable insight into the factors that interplay to control efficiency (e.g., Clauss *et* *al*., [39]). Snout shape is part of this suite of aspects of herbivore ecology that, combined, determine initial intake rate, chewing efficiency and forage selection ability [20], [40], [41], [42], [43]. Traditionally, a more pointed rostrum is associated with increased selection sensitivity, and a blunt rostrum is associated with a less selective cropping process with greater intake [24], [39], but these views remain in a state of partial flux [34], [44], [45]. Codron *et* *al*. [46] suggested that browsers and grazers vary their diets on a spatiotemporal scale, conforming to earlier studies by Owen-Smith and Du Toit [33 and 47]. Despite this variation, several distinctions have become apparent between browsing and grazing ruminant categories and are supported within a statistical and phylogenetic framework [17].

To date the association between anterodorsal snout morphology and feeding style has not been subjected to any geometric morphometric analysis of pure shape. Fraser and Theodor [43] demonstrated that anterior dentary shape (i.e., the ventral component of the snout) is strongly associated with diet. Furthermore, dentary shape was shown to be a good proxy for premaxilla shape and is strongly deterministic in selectivity during feeding. Such studies highlight the importance of controlling for phylogenetic similarity in tests of functional ecomorphology. We aim to build on such studies by using geometric morphometric techniques focussed exclusively on the dorsal snout, in particular the anterior section of the rostrum formed primarily by the paired premaxillae.

The principle aim of our study is to determine whether empirically assessed patterns of snout shape variation in ruminants support traditional distinctions that have been drawn between ‘browser’ and ‘grazer’ categories, and whether a quantitative geometry-based approach allows a more precise morphological definition of these functional categories to be formulated. The secondary aim is an assessment of the extent to which quantitative snout geometry may be used to predict the feeding styles in ruminants of ‘unknown’ or ‘intermediate’ feeding style, including fossil specimens for palaeoecology. The statistical null hypothesis under consideration is that snout profile shape exhibits no structured variation such that reliable morpho-functional categorisation is possible.

Furthermore, we investigate the influences of body size on snout morphology. Body size is an important ecological parameter in ruminants, affecting factors such as locomotion, digestive and ingestive efficiency [40], [48], [49], [50], competitive interactions [51], evolutionary and life histories [52]–[55], biogeography [56], [57], and sexual dimorphism [58]. Finally, phylogeny has been repeatedly demonstrated to be an important determinant of phenotypic aspects of ruminant life histories and ecology [59]–[62]. Accordingly, we explore the role of phylogenetic non-independence on snout morphology similarity using established comparative methods (e.g., [63]–[66]), and compare the results between a ‘raw’ geometric morphometric study, and one in which there is a measure of phylogenetic control.

## Materials and Methods

Geometric morphometrics involves the multivariate numerical analysis of two- or three-dimensional Cartesian coordinate data, typically defined by discrete, spatially-defined landmarks (i.e., topologically homologous loci on a structure [67]). Zoological studies are increasingly using a wide range of geometric morphometric techniques due to their intrinsic ability to summarize modes of variation in form – and so guide its interpretation – in many different systematic contexts [68], including functional morphology, sexual dimorphism, ontogenic development, and phylogenetic inference. The ruminant specimen-set analysed here consisted of 125 extant species, 119 of which were bovids or cervids as these are the most taxonomically diverse groups. Ecological categorisations were based on a number of sources and independent criteria, provided in Table S1. Categorisation authority was given to more recent studies where possible. Species traditionally classified as ‘intermediates’ or ‘frugivores’ were considered to be ‘unknown’ for the purpose of this analysis. Species with conflicting ecological classifications were additionally classified as unknowns, so that the browser and grazer categories used here were defined by species strictly characterised as browsers and strict grazers in the primary literature. The majority of sampled specimens were housed in the zoology collections at The Natural History Museum (NHM), London, UK, with additional specimens sampled from the Royal Veterinary College (RVC), London, UK. Specimen sex was not taken into account in calculation of the discriminant function, and, where multiple specimens were available, the largest were always selected to maintain consistency in age. The taxonomy follows the NHM labelling system, updated to conform to the species-level taxonomy of Fernández and Vrba [1]. It is assumed here that intraspecific shape differences are of a lower order of magnitude than interspecific shape differences; therefore, only a single specimen per species was used for the investigation.

Snout profile outlines were collected from photographs taken in ventral view with the crosshairs positioned centrally on the sagittal inter-premaxillary suture. The starting point for all the outlines was defined as the point where the suture between the maxilla and premaxilla intersects the left-lateral margin on the ventrolateral surface. This convention ensured that all subsequent semi-landmarks were interpolated to topologically homologous positions with respect to the total set of semi-landmarks used to represent the outline (each semi-landmark has a defined *x*–*y* position with respect to the co-ordinate system origin). One hundred equally spaced semi-landmarks were collected along each outline, a digitizing resolution sufficient to produce a geometrically faithful representation of the profiles. No semilandmark sliding was allowed for reasons discussed by MacLeod [69]. The raw, untransformed landmark co-ordinate data are provided in data file S1.

These semi-landmark data were subjected to a *Procrustes* (generalised least squares) transformation. *Procrustes* superimposition forms the core for analysis of pure shape by removing the extraneous variation in scale, orientation and position for all specimens’ semi-landmark constructions (see [70] and Box 2 of [71]). Optimising the fit of all specimens to each other was achieved by rigid rotation iteration until the distance between successive mean landmark configurations fell below 0.0001. This means that the analysis proceeded in shape space as opposed to form space. The specimens at this stage were sub-divided into their ecological sub-groupings for each subsequent analysis.

Superposed co-ordinate data for defined browsers and grazers were subject to a covariance-based principal components analysis (PCA) [72], which preserves the partial *Procrustes* distances among specimens. Three principal component (PC) axes accounted for 93.4 percent of the total shape variance (Table S1). Accordingly, projected scores on these three PC axes were retained and served as the basis for a secondary discriminant analysis. These principal component scores were then subjected to a canonical variates analysis (CVA) which, unlike PCA, includes the group-level information as an additional variable [73]. This multivariate technique transforms the data to a configuration that achieves the optimal discrimination between group centroids relative to the group dispersion structure [73]–[75] (S3). A log-likelihood ratio (LLR) test was performed to test group distinctiveness (i.e., the group dispersion structure) of these transformed data, with respect to the sample that defines the discriminant space [76]. The resulting probability estimate represents a validation test of the between-groups covariance structure; i.e., a low probability (<0.05, traditionally) reflects a statistically significant difference in the dispersion structure with respect to the defined groups.

Recently Mitteröcker and Bookstein [77] have questioned the use of CVA in geometric morphometric contexts as it is often the case in such datasets that the sample size is exceeded greatly by the number of variables used to represent form variation among the specimens in the sample (in this case, 200). To address this concern we applied Monte Carlo simulation and bootstrapping variations of CVA, each based on 1,000 pseudo-replicate datasets, to determine whether the between-groups distinctions observed are the product of hyper-dimensionality within the dataset [78]. The Monte Carlo simulation created pseudo-replicate datasets of values drawn randomly from a normal distribution of identical mean and variance to that of the pooled PCA scores. Each pseudo-replicate dataset was then subjected to a CVA and the extent of between groups discrimination summarized via calculation of the LLR index (*φ*) [78]. As all of the values used for the CVA were drawn randomly from the same distribution, this pseudo-replicate *φ* value represent the extent of-groups distinction expected under the null model of no difference between groups other than random sampling error. In addition, the set of 1,000 pseudo-replicate *φ* values was then be tabulated into a frequency distribution and used to assess the statistical significance of observed *φ* value obtained for the investigation dataset. The bootstrapping simulation created pseudo-random datasets drawn randomly from the PCA transformed raw shape data themselves such that the observed group structure was destroyed and, for each pseudo-replicate dataset random agglomeration of species of group sample sizes and variable numbers identical to the observed data were substituted. The distribution of random *φ* values derived from these bootstrapped pseudo-replicate datasets was then compared to the observed *φ* value obtained for the investigation dataset. Passing these sensitivity tests implies that the observed group distributions are the products of some extrinsic group-distinction factor (e.g., biogeography, phylogeny, functional constraints, ecology), as opposed being the result of random sampling or dataset dimensionality issues.

To represent a shape transformation sequence through the data based on hypothetical successive models of the snout profiles in both the principal component space, and a space defined by maximum between-groups shape variation, overlay or ‘strobe plot’ comparisons of modelled snout shapes were performed [79]. Three principal component axes, with five modelled points per axis, were back-projected into the space defined by the original *Procrustes*-transformed variables. These models represent the two extreme points, the central point, and two medially-interpolated points between these on each PC. The result is a set of non-orthogonal principal component axes oriented with respect to the data within *Procrustes*-scaled landmark data. Each modelled axis was plotted in order to assess, interpret, and illustrate the modes of shape variation represented along each PC axis. We repeated this procedure for the single discriminant axis produced by the CVA, and back-projected this into the PCA space to observe the major mode of between-groups shape variation in the space that defines the sum variation in sample snout shapes.

We additionally performed a test for phylogenetic signal in the principal component scores using Blomberg’s k, a commonly used statistic that is independent of sample size and assumes that a trait, in this case snout shape, evolved along a topology under Brownian Motion [80]. We also calculated Pagel’s λ which determines whether a structured or non-structured tree topology fits the trait data best [81]. Such practice is becoming increasingly common in ecomorphological analyses, and particularly within ruminants [49], [59], [60]. While several recent ruminant phylogenies exist for Cervidae [82], Bovidae [83] and all of Ruminantia [1], we opted to use the updated version of the Bininda-Emonds *et* *al*. mammalian supertree (M. Clauss, 2014, JPT, pers. comm., [84]), pruning both this tree and our data set so that taxonomic lists were congruent (n = 104, File S2). Blomberg’s k was calculated in the Picante package using the Kcalc() function, and Pagel’s λ in the Phytools package using the phylosig() function. We tested for phylogenetic signal using the residuals of a linear regression between body mass and the raw PCA scores. The results of this test prescribe whether or not the analysis needs to be repeated with a measure of phylogenetic control – in this case, using the phyl.pca() function in the Phytools package [85]. Body mass data were extracted from the PanTHERIA database [86] for all sampled species, and log-transformed subsequent to any analysis. All statistical tests were conducted in R v. 3.0.3 ([87]).

The process of dimensionality reduction, discriminant analysis, dispersion structure validation, and model visualisation provides a statistically rigorous protocol for assessing the validity of the ruminant feeding categories, and the morphology of the profiles that define these categories. Additionally, by exploring the effects of phylogeny and body size, we can determine if the pattern of shape variation exhibited by the sampled species is the product of evolutionary similarity, body size, an external ecological factor, such as feeding style, or a combination of all of the above.

## Results

### Principal Components Analysis

**Figure 1:**
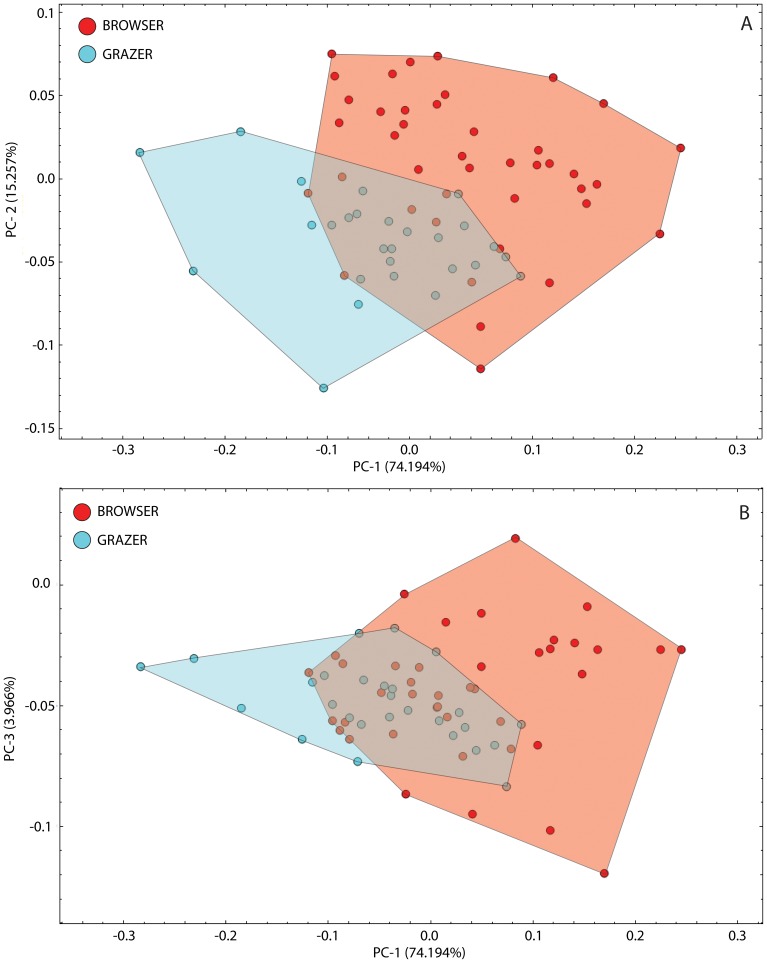
PCA score plots for browsing- and grazing-classed ruminants (A) PC-1 versus PC-2. The convex hulls represent a morphospace constrained by the extreme data points within the range envelope. Ecological classifications and PC scores for the species used to define this space are in Table S1. (B) PC-1 versus PC-3. The browser group occupies a similarly broad range as the PC-1 versus PC-2 plot, with grazers appearing more constrained along the PC-3 axis.

The first two principal component axes explained the overwhelming majority of the sample covariation (89.45 percent; Table S1). These axes can be used to define a low-dimensional shape ordination subspace (Fig. 1A), with an additional 4 percent of the covariance described by PC-3 (Fig. 1B). Grazer-classed species show less variation in this PC-1 and PC-2 subspace compared to browsers. The two groups overlap about the region of the total sample grand mean, but occupy quasi-distinct regions of the PC-1 to 3 subspace. Much of the morphospace occupied by grazing species in PC1–PC2 is defined by several outlier taxa (*Bos taurus*, *Connochaetes gnou*, *C. taurinus* and *Oryx leucoryx*), whereas the majority of this group occupy low negative scores about the grand mean. Grazers occupy more negative regions overall on PC-1 and browsers more positive values on both PC-1 and PC-2. Grazers are strongly constrained along the PC-3 axis, whereas browsers exhibit about twice the range variation in both the positive and negative values.

This dispersion structure implies that the dominant aspects of the shape covariance observed in the sample play a limited role in any between-group separation structure in the sample. Since PC-1 accounts for more than four times the variance as PC-2, this means that much of the shape variation present in the dataset is being determined by something other than distinctions between browsers and grazers, such as phylogeny, body size, or geographical partitioning. Overall the dispersion pattern of browsers is much sparser along PC-1 (defined at the positive-most extremity by *Alces pulmatus* and *Ammodorcas clarkei*, and negatively by *Beatragus hunteri*), whereas the distribution is much more constrained and defined by a higher density of species at extreme ranges on PC-2 (with the extremity defined positively by *Tragulus javanicus* and negatively by and negatively by *Rhynchotragus kirkii*).

**Figure 2:**
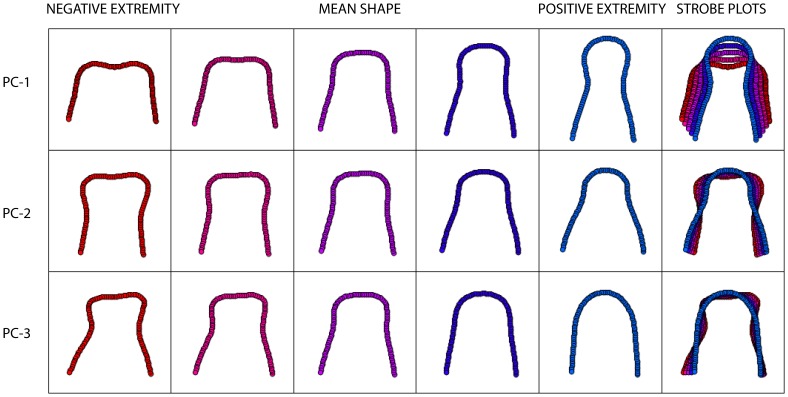
Strobe plots for the axis models associated with PC-1, PC-2 and PC-3. The right hand column is an overlay plot for each model series, showing the progressive deformation along each axis.

The between-groups shape deformation axes were modelled at five coordinate positions along the first three PCA axes (Fig. 2). As can be seen from these models, positive scores on PC-1 are associated with profile shapes exhibiting an elongated rostrum (approximately twice the length compared to the maximal width of the premaxillae), with a convex distal end, and a distinct medial compression at the mid-point of the lateral premaxillae margins. Negative PC-1 scores describe premaxillae that are slightly wider than long, with a distal medial concavity and lateral margins that gradually diverge posteriorly. The anterior end of this model profile is slightly concave. This represents a continuous transformation of lateral broadening and longitudinal contraction from positive to negative score values. This difference in muzzle length between different feeding categories was first noted by Fraser and Theodor [43]. Positive scores on the PC-2 axis describe a sub-triangular geometry, with a convex distal end. The lateral margins of these profile shapes diverge rapidly posteriorly, with a slight lateral contraction that is not distinct as in PC-1. Negative PC-2 scores describe mediolaterally compressed ‘club-like’ shapes, with a slight anterior concavity similar to PC-1. This axis represents a shape deformation sequence in which the posterior part of the lateral premaxilla narrows in width, and the anterior part expands laterally but compresses longitudinally from positive to negative PC-values. The major mode of change described by PC-3 is from a distally flattened rostrum with strongly laterally convex margins (negative scores) to a distally convex rostrum (positive scores), with a slight component of asymmetrical vergence in the higher values. This pattern of deformation is unusual in that it is occurring in a non-symmetrical mode about the sagittal line of the rostrum. It is likely that this axis is detecting a portion of heterogeneous shape change associated with deformation not removed by the *Procrustes* transformation, and perhaps due to a small degree of warping in the premaxillae from drying involved in the collection and storing process. As this mode of deformation is represented by such a small proportion of the sample variance, we do not consider this to be a major problem with our samples.

### Canonical Variates Analysis

**Figure 3:**
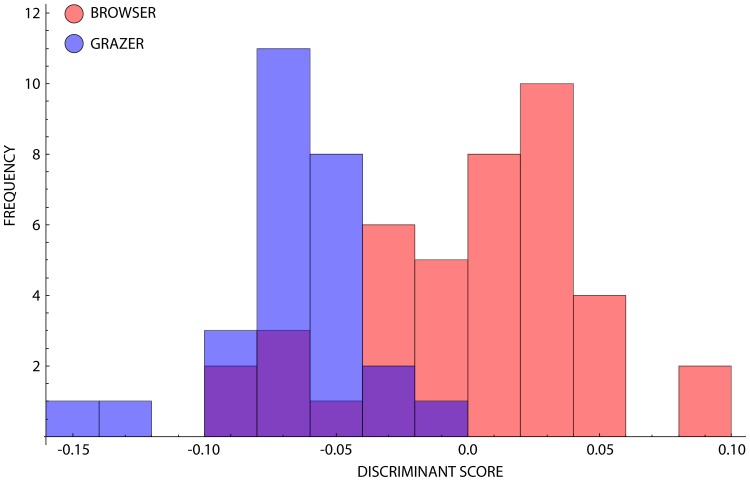
Histogram showing the frequency of occurrences of browser-class and grazer-class species along CV-1. The occupation of quasi-distinct discriminant spaces is clear, with overlap about the grand mean.

The first three PC axes accounted for the majority of the total observed sample shape variance (93.42 percent). Scores of individual semi-landmark shape configurations on each of these axes were parsed into grazers and browsers based on lines of evidence independent of snout morphology and subjected to a CVA. Since only two groups were used for this analysis, a single discriminate axis was defined. A histogram of results for the projection of shape configurations onto this axis is given in Figure 3. Both browsers and grazers occupy relatively broad regions with grazers occupying negative values along CV-1 whereas browsers are distributed more positively (see Table S2 for associated CVA scores).

The overlapping ranges of browsers and grazers implies that the within-groups shape variation is distributed in a manner such that a complete snout profile continuum exists irrespective of whether the shape space is formulated to reflect the major axes of pooled groups variance (Figs. 1A, B) or between-groups distinction (Fig. 3). Nevertheless, quasi-distinct regions within the discriminant space can still be identified. A log-likelihood ratio test confirmed this distribution as not being the product of random variation within the dataset (p<0.0001). Monte Carlo simulation and bootstrapping variations of CVA both based on 1,000 pseudoreplicate datasets produced distributions of randomized LLR (*φ*) values that were all well below that of the empirical data (*φ* = 38.13; p = 0 in both circumstances). This indicates that the probability of these groups occupying their positions in the overall CV space as a result of the effect of random sampling of a single, underlying population is well below the traditional 95 percent level of statistical significance. Accordingly, the alternative hypothesis – that the observed magnitude of centroid separation is such that these data were most likely drawn from different shape populations with different characteristics – is supported. Although these snout outline shapes are distributed continuously between ruminant species, there is an underlying trend driven by the different group-based ecological categorisation of these profiles.

Proximity estimates are provided by calculation of a confusion matrix (Table S2), which summarizes the assignment of species with respect to their *a priori*-defined groups based on their distances to the respective group means in the canonical variates space. This result indicates that in over four out of every five cases (83.82% for this dataset), the correct *a posteriori* assignment of each species to its *a priori* designated feeding class, based on secondary criteria, was possible using premaxilla shape data alone. As such, the division of ruminants in to broad, but non-exclusive, categories of browsers and grazers maintains a high level of statistical support. A jackknifed (leave one out) cross-validation of the performance of this discriminant space produced results similar to the original CVA, with 83.58% of correct *a posteriori* assignment with just a single grazer being identified incorrectly.

**Figure 4:**
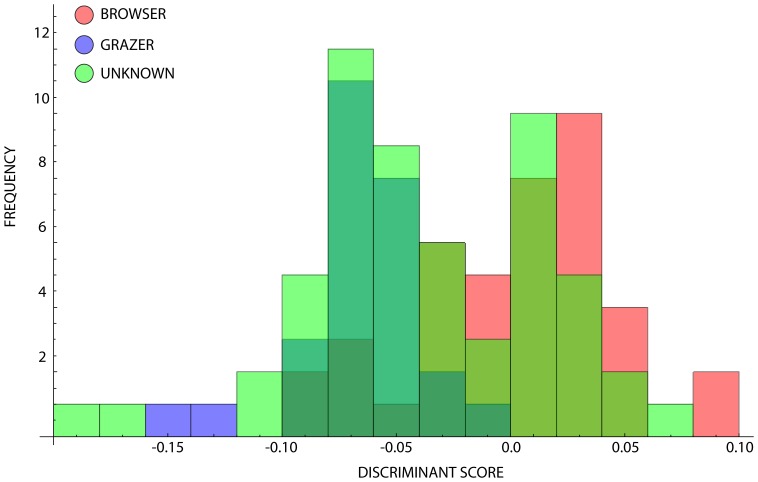
Histogram plot for ruminants classified according to their feeding strategy, with ‘unknowns’ projected into the space. The unknown-classed species’ range plots more negatively on the CV-1 axis, suggesting that there is a ‘cryptic’ measure of snout shape variance that is not picked up in the traditional browser-grazer dichotomy.

The ‘unknown’ sub-group was projected into this defined space as a way of indicating which of the known groups they belong to, and as such what ecological inferences can be made about them with a confidence of 83.82 percent (Fig. 4). By calculating the distance from each projected point to the known group centroids, we were able estimate the likely candidate group to which these ‘unknown’ and ‘uncertain’ species belong. Of the 57 unknown species, 32 are assigned provisionally to the grazer category, and 25 to the browsers. These provisional assignments can be validated by using additional observational data, such as the percentage of grass consumed, or the hypsodonty index, once these are rigorously defined as being able to discriminate between the different feeding styles. It is however worth noting that the assignment of several of the taxa that fall outside of any of the known browser- or grazer-defined spaces cannot be justified based on the current analysis.

**Figure 5:**
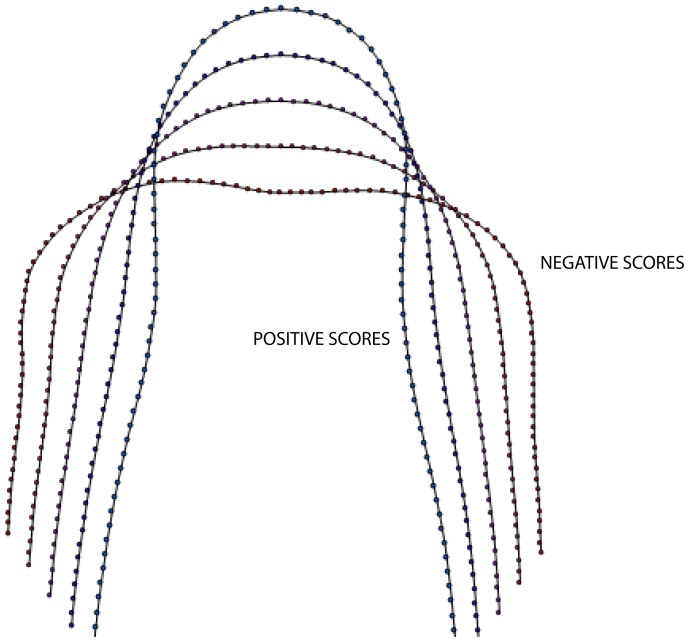
Overlay plot of the CV model axes in PC space for browsers and grazers showing the progressive geometric deformation between modelled axis points. The general profile change is from blunt to pointed, but this excludes some of the subtle profile shape changes.

To interpret the geometric character of optimal between-groups shape discrimination, the single CV axis was modelled at five equally-spaced coordinate positions as with the PC axes. The shape configurations present at these positions along this CV axis were determined in a manner similar to that used to create the PC models (see above), first by back-projecting them into the corresponding PC-space and then reconstructing the semilandmark point configurations at those positions using the method of MacLeod [78], [79] (Fig. 5). The pattern of shape variation described by this CV axis incorporates all three of the modes represented by the PC axes described above. It can be regarded as a continuum that shows progressive deformation of the premaxilla from a rostrolaterally broad, moderately laterally convex, and distally depressed geometry (negative scores) into a laterally convex and posteriorly divergent, and distally narrow and pointed shape (positive scores). This model ‘strobe plot’ provides a more explicit, detailed, and empirically-based visualisation of this broadly pointed-to-blunt shape continuum. The fact that these discriminant axis models are almost identical to those of PC-1, the major axis of shape variation, implies that the shape distinction between browsers and grazers is more significant than any other factor in reflecting the current state of ruminant snout morphological diversity.

### Comparison with body mass and phylogeny

We used the Bininda-Emonds *et* *al*. supertree [82] pruned to include only those taxa that are present in the current sample dataset (n = 104). Using the residuals from a linear regression model of the first two PC axis scores against body mass, we derived Pagel’s λ as a measure of phylogenetic signal (PC-1, p = 0.014; PC-2, p = 0.008). We additionally derived the Blomberg’s k statistic as a comparison, finding non-significant results for the residuals for both major PC axes (PC-1, p = 0.228; PC-2, p = 0.192). Taken as a whole, however, these results suggest a significant component of phylogenetic signal within these shape data. Accordingly, we calculated independent contrasts for the body mass data and scores for the first two PC axes. A Spearman’s rank (p = 0.8613, ρ = 0.017) and Kendall’s tau test (p = 0.934, τ = 0.006) demonstrates that there is no significant relationship between body mass and PC-1, the primary axis of snout shape variation, within the ruminant data set when phylogenetic independence is controlled for. Similar results were obtained for PC-2 for both Spearman’s rank (p = 0.445, ρ = −0.076) and Kendall’s tau (p = 0.454, τ = −0.05).

**Figure 6:**
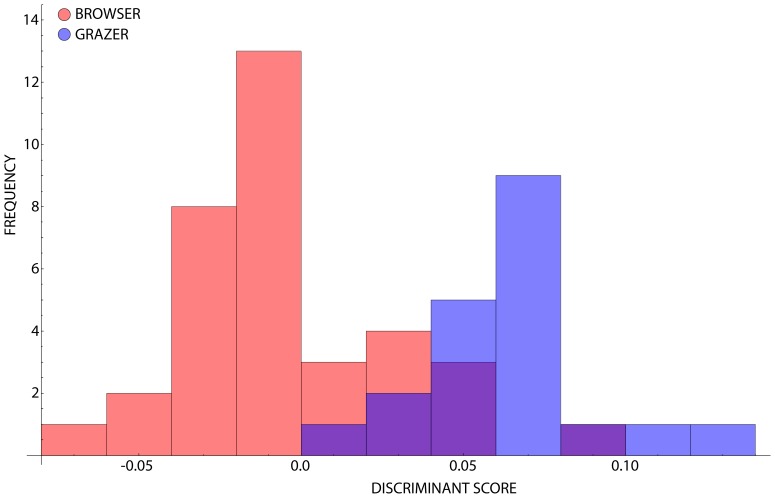
Histogram showing the frequency of occurrences of browser-class and grazer-class species along CV-1, based on a canonical variate decomposition of the phylogenetically controlled principal component scores (PC1, PC2, and PC3). Note that the frequency distributions for each group appear to be superficially taking on that of a normal distribution about different means.

Due to the significant phylogenetic signal in the principal component scores, we modified and repeated the previous multivariate analysis procedure using the raw shape variables by performing a phylogenetic PCA on the superimposed *Procrustes* co-ordinates [85]. The results of this extended phylogenetically controlled analysis are given in Table S3. The resulting canonical variates histogram produces similar results to the raw analysis, with overlapping but quasi-distinct discriminant spaces occupied by both browsers and grazers (Fig. 6). It is important to note that the frequency peaks of these distributions are distinct from each other in this space, falling either side of the mean shape. The confusion matrix indicates that the phylogenetically controlled discriminant analysis performs slightly better than the non-controlled analysis in correctly resolving individual species to their category, with 85.45% correct assignments. The jackknifed confusion matrix confirms the stability of this distribution, with a slight reduction to 85.19% correct assignment. The log-likelihood ratio test of this distribution is strongly significant, (p<0.0001), and the stability confirmed as before with bootstrapping of the distribution (p = 0.0) and Monte Carlo simulations (p = 0.0).

**Figure 7:**
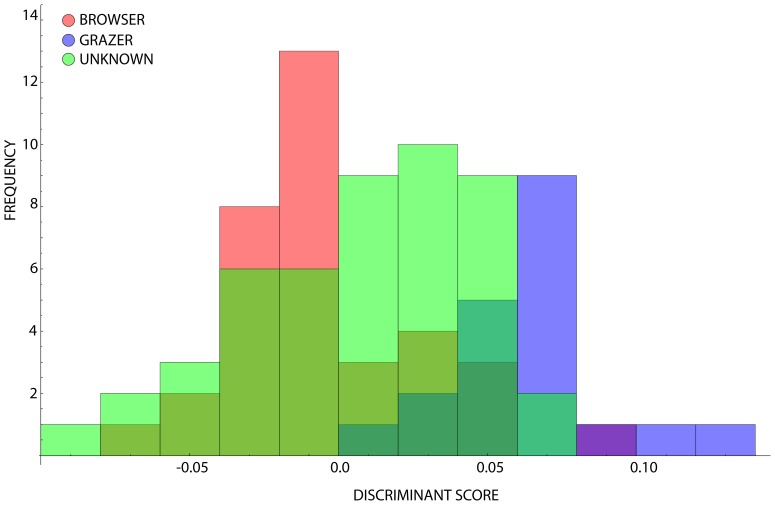
Histogram plot for ruminants classified according to their feeding strategy with ‘unknowns’ projected into the phylogenetically controlled CV-1 space. Note the high frequency distribution about the grand mean of the browser-grazer defined space.

Projecting the ‘unknowns’ into this discriminant space as in the raw analysis shows that they overlap both group spaces pervasively, and exceed the browser space on the negative CV-1 axis (Fig. 7). Of the 48 ‘unknown’ species in this slightly reduced dataset, 31 (64.58%) are assigned to the browser group, and 17 to the grazer group (35.42%), which based on the assessment purely with browsers and grazers we can state with approximately an 85% confidence level (Table S3). The overall result is that controlling for phylogenetic similarity in these tests is not sufficient to change the stability of the resulting group dispersion structures, despite their being a high degree of phylogenetic signal in the shape profiles within the sample dataset.

## Discussion

Taken as a whole, our results suggest that snout shape is largely sufficient to differentiate between - and so to identify - different feeding styles in ruminants. Initial descriptions of the blunt-pointed dichotomy do indeed represent an aspect of the deformation sequence and describe it in a simple way. While the results of this study largely confirm that of previous research (e.g., [24], [36], [37], [42]), the approach used herein gives analysts access to the total range of shape variation expressed by geometric morphometrics, and so provides a greater quantitative appreciation of the complexity of the shape transformation. It is apparent that ruminants are so morphologically diverse, and have adapted to maximise resource exploitation in their respective ecosystems to such an extent, that they form a continuum of shape variation between ‘browser-type’ and ‘grazer-type’ end members. Such a result might be explained by ‘constrained divergence’, whereby the two groups’ rostrum shapes were originally quite similar, but have diverged over evolutionary time.

Previous work assessing the relationship between snout shape and diet (e.g., [36], [42]) has largely followed the methodology of Walker [88], using it primarily to aid for inferring the diets for extinct ruminant species. These assessments were based on quantitative interpretation of exemplar taxa, with the method requiring construction of the anterior dorsal snout curve using a cubic spline-fit function to assess intraspecific variation. Typically, authors have selected the 26 degree angle for the whole sample to reconstruct the angle of lateral intersection from the midline, a seemingly arbitrary decision which may not be a consistently strong indicator of snout shape for each specimen. More recent research, such as the comparative study by Fraser and Theodor [43], extended these earlier studies by comparing the utility of numerous snout shape metrics in reconstructing diet, additionally incorporating information from the anterior dentary. We have demonstrated that, using geometric morphometrics, such linear morphometric approaches are largely sufficient in capturing the complexity in shape variation of ruminant snouts. Other authors have identified snout width as a proxy for distal snout shape, with measurements taken at the ventral maxilla-premaxilla intersection on the lateral margin [24], [37]. When describing the geometry of complex shapes a single linear metric is usually inadequate as equivalent measurement values can describe completely disparate geometries of varying complexity, and non-comparable function. These authors used this type of measurement, along with the palatal length, to define a ‘relative muzzle width ratio’, which they used to represent the ratio between body size and the oral aperture, as well as possibly representing oral intake and processing rate. Ratios are poor shape descriptors since all a ratio can represent adequately is an ellipse, if the two measurements represent orthogonal axes, as in the method used by Solounias and Dawson-Saunders [89]. This approach may be sufficient for partially representing extremes of the browser end of the shape spectrum, but can just as easily describe a typically blunt grazing form. The simple fact is that the set of shapes the same ratio can represent is infinite. Hence, ratios can be inappropriate tools for snout shape characterisation (contra [24]). However using linear morphometrics, Fraser and Theodor [43] find a high rate of dietary classification, in particular when the intermediate or mixed feeders are excluded from analyses. The implication of this result is that while outline morphometric methods can describe the complexity of shape variation and confirm the presence (or absence) of shape differences in a more geometric manner, linear distances may be adequate for encapsulating the same amount of ecological information from ruminant snouts, and arguably in a more efficient manner.

The principle focus of this study was to determine whether ruminant snout profile shapes form discrete varieties that covary with feeding strategies (assessed via independent evidence) as had been suggested by numerous previous studies [24], [36], [37], [42]. The corresponding null hypothesis relates to the conclusions of Pérez-Barbéria and Gordon [29], among others, that feeding strategy bears no precise relation to premaxilla morphology. One alternative hypothesis is that the shapes of ruminant premaxilla form a continuum, with characteristic ‘browser-type’ and ‘grazer-type’ morphologies comprising end-members, a hypothesis that is increasingly winning support based on a range of detailed investigations. This hypothesis is based on the inference that classifying what are intrinsically morphologically diverse organisms into discrete clusters is problematic and somewhat counter-intuitive, if purely for the purposes of having an antecedent framework onto which new hypotheses of functional morphology can be built. Our results show that, when ruminants are classified ecologically as browsers and grazers based on a range of secondary criteria, they cannot be discriminated completely based on the shape of their premaxillary profile. This result is inconsistent with previous investigations of this issue in which this shape dichotomy was assumed to be absolute based on the sample species used [24], [36], [37], [42]. Premaxilla shape appears to be moderately homoplastic in nature, with a broad range of profile geometries being present in both of the feeding-style sub-groups. Despite exhibiting a degree of shape overlap, these groups retain moderate geometric independence, such that they can be assigned to the correct groups post hoc over 80 percent of the time. While profile-based classification among ruminants is not perfect, it nevertheless has potential to inform studies of fossil ruminants, as it enables quantitative assessment of inferring their ecologies as well as providing a means of quantifying the statistical confidence that can be assigned to these inferences.

The results obtained by our investigations also suggest a possible route of analysis that can be employed in future investigations of functional ecology in ruminants: specifically the use of multivariate ordination analysis combined with tests of statistical confidence to assess the validity of naturally-occurring groups. A similar conclusion was reached by Pérez-Barbería *et* *al*. [90] that currently accepted boundaries between ruminant feeding strategies remain somewhat arbitrary. One approach to resolving this problem would be to employ a covariate or group of covariates as continuous variables, with thresholds being based on the identification of functionally significant and discrete clusters. However, investigations into this issue so far have found no morphological discrepancies that can explain variation in ruminant digestive efficiency based on digestive, not ingestive, morphology [2], [19], [32], [91]. This perplexing result may, in part, be due to the treatment of species as static entities, when realistically thresholds should be constructed on a sliding scale accounting for population-level ecological, environmental and spatiotemporal variations where appropriate [33], [35]. Interpretation of general patterns must also be flexible enough to account for singular exceptions (e.g., frugivores) and are currently insufficient to encapsulate the full diversity of ruminant feeding habits.

Theoretically, a higher food intake rate should covary with the evolution of stronger anatomical structures [92] (e.g., strengthening or fusion of sutures, increased muscle attachment area, decreasing pleurokinesis and increased resistance to strain). This relationship between diet and ecology does not necessarily imply that as snout shape, and hence intake rate, varies, it forces covariation of other morphophysiological parameters. Rather, snout shape constitutes an initial parameter with which other functional domains interact. This morpho-functional relationship was corroborated by Fletcher *et* *al*. [93], who proposed that the strength of the masticatory apparatus has a functional or adaptational origin, challenging other studies which identified it as being a phylogenetic artefact [29], [37], [94], [95]. This covariation hypothesis requires further investigation, with snout shape being analysed to assess functional significance as a trait affecting both intake rate (volume per unit of time) and selectivity (non-parametric), and plausibly maximum bite size (volume) [96], [97]. The results herein imply that snout shape, and thus feeding style, has a strong adaptive component combined with phylogenetic constraint, based on the analysis of a broad range of ruminant species. Finally, our results suggest that major variations in snout shape are related to body size variation, although a directional relationship cannot be established.

## Conclusions

Using a two-dimensional representation of the ruminant snout in ventral aspect, we have demonstrated that there is a strong relationship between snout shape and feeding ecology within a highly diverse sample of the major ruminant clades, but only when the data set is restricted to members of the relatively well-defined browser and grazer classes. This between-group discrimination is statistically robust, and supported by recent analyses of the relationship between diet and the shape of the anterior dentary [43]. Snout shape variation is shown to be strongly controlled by phylogenetic similarity, but with this phylogenetic component not affecting the overall dispersal patterns of snout shapes in discriminant space and proportion of successful categorisations. Snout shape variation is also found to be strongly correlated to body size, although this relationship breaks down in a phylogenetically controlled comparison. This corroborates previous hypotheses of relations between feeding style, body size, and ecology, and that while evolutionary similarity is an important component of ecology, snout shape appears to reflect a genuine functional signal.

Based on our results, it is further apparent that previous categorisations, which included putative ‘intermediates’, or ‘unknowns’ here, of snout shapes relative to feeding strategy are not fully adequate in their depictions of the full range of exhibited morphological variation (i.e., ‘browsers’ do not strictly have ‘pointed’ premaxillae, and ‘grazers’ do not just have ‘blunt’ premaxillae as asserted previously by many authors). The geometric complexity of premaxilla morphology is more extensive than this and forms a continuum of shape variation within the modern ruminant fauna. Our results suggest that attempts to place thresholds on other related factors involved in feeding are problematic and quantitative testing is required *a priori* (following the recommendations of Gordon and Illius, [34]).

In light of these results, inferences made by Janis *et* *al*. [92] - that intake rate forces covariation in the anatomical strength of the mandible - could be explored further to determine the relationship between grazing and browsing ruminants and the relative robustness of the masticatory apparatus. We suggest, in a manner analogous to that of Codron *et* *al*. [46], that ruminant diets represent a continuum with variation explicitly occurring on a spatiotemporal (geographical and seasonal) scale for all feeding strategies. This requires additional analysis in terms of ruminant phylogenetic affinity, [98], [99], [100], species’ ranges, and functionally significant ecological parameters. Additionally, the role of different ecological categories based on dietary strategies could be explored beyond the traditional browser-intermediate-grazer trichotomy, such as that for African bovids by Gagnon and Chew [101].

The fact that feeding style-based categories were demonstrated to be associated with snout shape in this investigation offers a model for future ecological studies regarding the reconstruction of palaeodiets using a morphometric dataset to delimit and identify extinct browsing and grazing species [36], [38]. This aspect of palaeoecology could feasibly be integrated with additional indicators of diet, such as isotopic signatures and microwear in teeth [99], [100], [102], or the hypsodonty index [103]. Indeed, the incorporation of additional ecological predictors has been demonstrated to increase the accuracy of dietary classification [42].

It is conceivable that our results are the product of a lack of consistency in the ecological definitions of functional feeding groups for ruminants – either in theory or in practice – with respect to other morphophysiological traits. The functional significance of snout shape in relation to bite size, intake rate, body size and selectivity was not addressed explicitly by our investigation. Indeed, our results indicate that closer inspection of these relationships is required. Quantitative metrics describing these ecologically significant parameters should provide a firmer basis for these in (anticipated) future studies [97].

What is undoubtedly necessary in future studies is the dissection of recovered signals to determine what proportion of trait covariation can be explained by phylogenetic relationships [94], [95]. Applicable methods include comparative phylogenetic modelling, which has gained increasing interest in the integration of ecology and macroevolution (e.g., [95], [104]), and demonstrated in the current study in the context of multivariate statistics. This will facilitate the teasing apart of genuine adaptational signals as opposed to morphological similarity based on common ancestry. Calculation of the rates and direction of snout shape evolution in ruminants, while incorporating fossil data, will be important in elucidating the ecological history of ruminants. Furthermore, if singular or multiple functional traits are found to be phylogenetic artefacts, it may be possible to track the sequence of acquisition, and therefore trace the macroevolutionary and ecological coevolution of ruminants. However, the results obtained here suggest that while phylogeny exhibits a strong control on snout shape in ruminants, it does not affect their ecological classification. Indeed, snout shape and profile-based classification can be explained by a combination of phylogenetic similarity and evolutionary history, body size, and ecology. Finally, in addition to phylogeny, factors such as ontogeny and range size should be scrutinised within a similarly rigorous morphometric-statistical framework to detect potential allometric variation, possible synchronisation of trait acquisition, and evolutionary patterns of character acquisition that might differ between sexes.

## Supporting Information

Table S1 Categorical data used for all analyses, PCA eigenvalues, PCA scores, PCA eigenvectors, and body sizes.
(XLS)

Table S2 CVA scores, confusion matrix, jackknifed confusion matrix, browser and grazer distance table, and the unknown projection distance table.
(XLS)

Table S3 Results of the phylogenetically controlled component of this study, including PCA scores, CVA scores, browser and grazer distance table and confusion matrix.
(XLSX)

File S1 **Raw co-ordinate data for all 125 ruminant species.**
(TXT)

File S2 **Ruminant phylogeny used for comparative analysis.**
(TXT)
